# The metabolic escape: how tumor metabolic reprogramming drives drug resistance

**DOI:** 10.7717/peerj.21400

**Published:** 2026-06-08

**Authors:** Yan Chen, Zixu Wu, Wenzhe Si, Xujun Liu

**Affiliations:** 1 Department of Laboratory Medicine, Peking University First Hospital, Beijing, China; 2 Department of Laboratory Medicine, State Key Laboratory of Vascular Homeostasis and Remodeling, Peking University Third Hospital, Beijing, China

**Keywords:** Cancer, Metabolic reprogramming, Drug resistance, Tumor microenvironment, Cancer therapeutics

## Abstract

In the last few years, metabolic reprogramming has been recognized as a fundamental characteristic of cancer, and is also acknowledged as a crucial cause to drug resistance, which consistently acts as a significant barrier in cancer treatment by allowing tumor cells to adapt and escape various therapies. This review gives a systematically investigation of how metabolic reprogramming contributes to drug resistance in cancer, including aerobic glycolysis (also known as the Warburg effect), lactate metabolism, glutamine addiction, lipid synthesis reprogramming, mitochondrial and ion metabolic changes. Furthermore, by clarifying the mechanisms behind these reprogrammed metabolic pathways, we explain how these changes lead to drug resistance and highlight potential molecular targets for therapeutic intervention. Additionally, we discuss emerging strategies aimed at exploiting these metabolic vulnerabilities, offering new insights for overcoming drug resistance in cancer. By integrating recent discoveries in this field, we present a unified perspective on targeting metabolic vulnerabilities to overcome drug resistance, which is an urgent need in precision oncology, and timely and concise insights for cancer biologists and researchers in the field of exploring the metabolic mechanisms of drug resistance. We hope this review will provide valuable insights for molecular tumor biologists seeking to elucidate the molecular roles of tumor metabolic reprogramming and drug resistance in cancer.

## Rationale

Cancer remains a leading cause of mortality worldwide, with drug resistance representing a major therapeutic obstacle. Metabolic reprogramming has emerged as a fundamental hallmark of cancer and a pivotal driver of this resistance.

This rewiring of cellular metabolism allows cancer cells to sustain proliferation, adapt to stress, and evade therapy. Furthermore, metabolic heterogeneity and the tumor microenvironment (TME) critically contribute to resistance; cancer stem cells utilize distinct metabolic pathways, and stromal cells secrete metabolites that protect tumors. We systematically explores how core reprogrammed pathways, including aerobic glycolysis, lactate metabolism, glutamine addiction, lipid synthesis, and alterations in mitochondrial and ion homeostasis. By integrating recent advances, we aim to highlight the mechanisms underpinning this metabolic escape and discuss emerging therapeutic strategies designed to target these vulnerabilities, offering new avenues to overcome resistance in cancer treatment.

## Introduction

**Figure 1:**
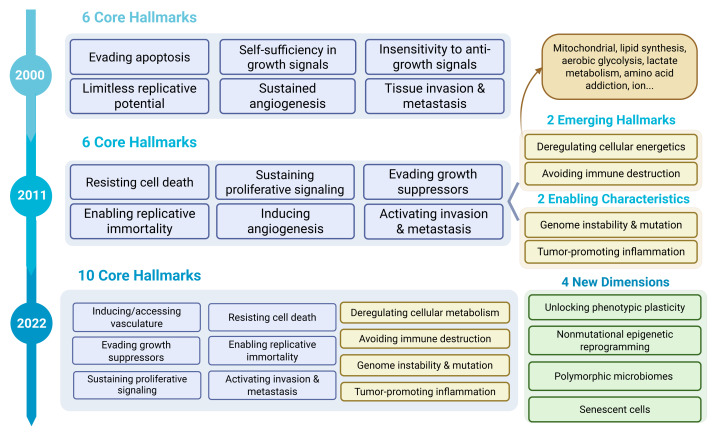
The conceptual evolution of the hallmarks of cancer and the pivotal role of metabolic reprogramming. The conceptual framework of cancer hallmarks has significantly evolved since its inception. Originally encompassing six core capabilities in 2000, the roster expanded in 2011 to include emerging hallmarks and enabling characteristics. In the 2022 update, the framework has been further refined into a multidimensional landscape comprising ten core hallmark capabilities and four new dimensions, reflecting the increasing complexity of tumor biology. Notably, several hallmarks have been renamed or redefined to encompass broader mechanisms. In this review, we specifically highlight deregulating cellular metabolism as a central pillar of tumor progression. The arrows originating from this hallmark indicate its key functional manifestations and consequences, such as aerobic glycolysis and amino acid addiction, which collectively synergize to drive tumor adaptability and the development of drug resistance.

Cancer remains a leading global health burden, with close to 20 million new cases and 9.7 million deaths reported worldwide in 2022 alone (Bray et al., 2024). Despite advancements in early detection and therapeutic innovation, drug resistance continues to undermine clinical outcomes, leading to high mortality rates across diverse malignancies. The hallmarks of cancer, originally defined as six core capabilities, have evolved into a more complex multidimensional framework (Hanahan & Weinberg, 2000; Hanahan & Weinberg, 2011) (Fig. 1). Notably, deregulating cellular metabolism has transitioned from an emerging characteristic to a core hallmark (Hanahan, 2022). This metabolic shift is no longer viewed merely as a metabolic byproduct of rapid growth, but as a fundamental mechanism that enhances tumor adaptability and drives the development of drug resistance. Metabolic reprogramming changes cancer cells by replacing metabolic programs that exist in most normal tissues, and enables cancer cells to rewire energy production, biomass synthesis, and redox homeostasis, all of which contributes to cancer’s uncontrolled proliferation, environmental adaptation and drug resistance. For instance, under oxygen deficiency and other conditions, some hepatocellular carcinoma cells (HCC) have a preferential activation of AKT/PKB and Bcl-2 cell survival response, which conferring chemo-resistance (Deshmukh et al., 2016; Lei et al., 2023). Similarly, colorectal cancer (CRC) uses enhanced glycolysis and altered glutamine metabolism to bypass oxidative phosphorylation (OXPHOS) by shifting toward the Embden-Meyerhof-Parnas (EMP) pathway, thus fostering resistance to chemotherapy and radiation (Pang & Wu, 2025). These adaptations underline the central role that metabolic reprogramming plays in promoting tumorigenesis and evading therapy.

The interplay between metabolic heterogeneity and the tumor microenvironment (TME) further complicates resistance mechanisms. Cancer stem cells (CSCs), characterized by their reliance on glycolysis and glutaminolysis, exhibit intrinsic resistance to conventional therapies by maintaining quiescence and activating drug efflux pumps (Deshmukh et al., 2016). Stromal components, such as cancer-associated fibroblasts (CAFs), secrete metabolites like lactate and ketone bodies, creating a nutrient-rich niche that shields tumors from cytotoxic insults (Mir et al., 2025; Zhu et al., 2022). Spatial and temporal heterogeneity in metabolic pathways ensures that subpopulations of cells survive treatment, leading to relapse and resistance (Dagogo-Jack & Shaw, 2017).

This review aims to systematically unveil the pivotal role of metabolic reprogramming plays in drug resistance. We choose some critical pathways in cancer development, including aerobic glycolysis (also known as the Warburg effect), lactate metabolism, amino acid addiction, lipid synthesis, mitochondrial and ion reprogramming, and corresponding strategies for those targets. In the following context, we first conclude how cancer cells exploit different metabolic pathways to evade conventional and targeted therapies, and then we explore emerging therapeutic strategies designed to target these metabolic vulnerabilities, thereby counteracting resistance. By illustrating these mechanisms and integrating these insights, we highlight the urgent need for integrating metabolic therapeutics into precision oncology to reduce drug resistance and so improve treatment efficacy and patient survival rate.

## Survey Methodology

We conducted a systematic literature search using PubMed, a comprehensive database providing extensive access to peer-reviewed scientific publications, thereby ensuring broad coverage across disciplines relevant to cancer biology and pharmacology. An iterative search strategy was applied. Initially, literature related to cancer metabolic reprogramming and drug resistance was retrieved to identify key thematic areas. Based on these themes, targeted searches were subsequently conducted for each specific aspect discussed in this review. The following search terms were employed: “drug resistance”, “cancer,” and “metabolic reprogramming,” combined with appropriate Medical Subject Headings (MeSH) terms or free-text keywords as applicable. The primary literature search focused on articles published between 2015 and 2025, with a limited number of earlier studies included when they were considered foundational to the field. Inclusion criteria: articles were selected if they provided detailed insights into the mechanisms of metabolic reprogramming in cancer, with an emphasis on studies published in peer-reviewed journals, including original research articles and authoritative review papers. Exclusion criteria: studies were excluded if they lacked rigorous experimental design, sufficient methodological detail, or clear, data-supported conclusions. Screening process: titles and abstracts were reviewed to ensure relevance, followed by a detailed examination of the full text. The screening and final selection of articles were conducted by the authors.

### The metabolic landscape of the tumor microenvironment

The tumor microenvironment (TME) is a distinct spatial domain with unique physicochemical properties. While the microenvironment of normal tissues typically maintains stable oxygen supply, a neutral-to-slightly alkaline pH, and equitable nutrient distribution, the TME is characterized by high complexity, heterogeneity, and extreme conditions (Dzobo, 2020). This uniqueness is primarily manifested in its physical attributes: due to malformed and dysfunctional tumor vasculature, the TME is subjected to chronic hypoxia, accompanied by profound acidosis where pH often reaches acidic levels (frequently below physiological pH), resulting from the accumulation of metabolic byproducts, as well as elevated interstitial fluid pressure (IFP) caused by impaired lymphatic drainage. These extreme physicochemical features not only create a physical barrier to the penetration of chemotherapeutic agents but also force many cells within the niche to undergo metabolic reprogramming for survival (Liu et al., 2024; Nicolini & Ferrari, 2024).

In terms of composition, the TME is far more than a mere collection of cancer cells; it is composed of interwoven cellular and non-cellular components (Dzobo, 2020). The cellular compartment primarily includes cancer-associated fibroblasts (CAFs), the most abundant stromal cells that construct the structural scaffold and metabolic reservoir of the tumor; a diverse immune cell population, comprising effector components such as T cells and natural killer (NK) cells, as well as pro-tumorigenic components like regulatory T cells (Tregs), myeloid-derived suppressor cells (MDSCs), and tumor-associated macrophages (TAMs); and vascular endothelial cells and pericytes (Wang et al., 2024b; Zhang et al., 2023). The non-cellular compartment consists of a complex extracellular matrix (ECM), various cytokines, chemokines, and exosomes, which collectively form a dynamic signal transduction network (Dzobo, 2020).

**Figure 2:**
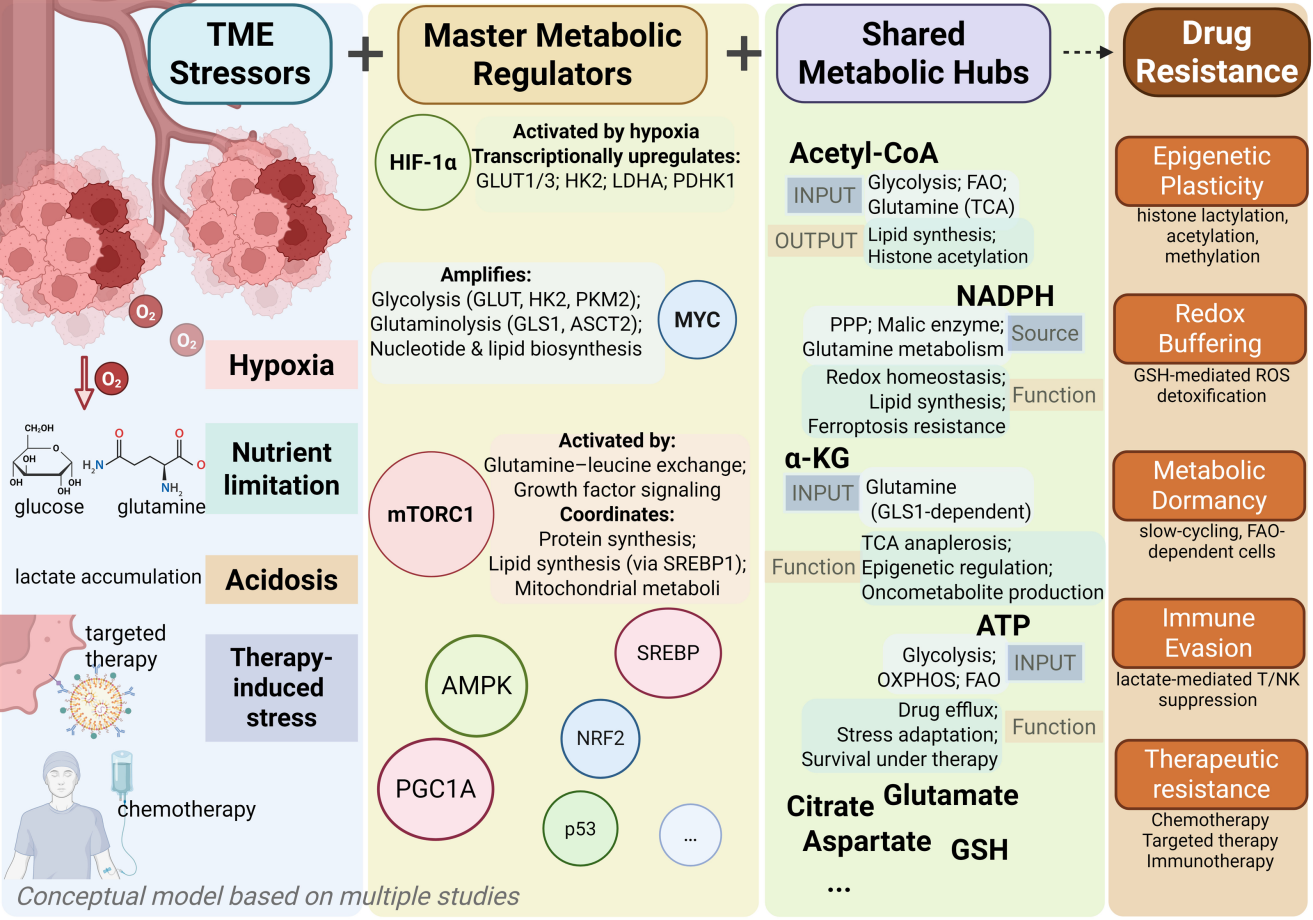
A proposed conceptual model of cross-pathway metabolic integration in the tumor microenvironment (TME) driving drug resistance. Findings from multiple independent studies across different cancer types and experimental systems (does not represent data from a single empirical study). The TME imposes multiple metabolic stresses, including hypoxia, nutrient limitation, acidosis, and therapy-induced selective pressure. These conditions activate central metabolic regulators such as hypoxia-inducible factor-1*α* (HIF-1*α*), MYC, and mechanistic target of rapamycin complex 1 (mTORC1), which coordinately reprogram cancer cell metabolism. Rather than acting as isolated pathways, glycolysis, glutamine metabolism, lipid metabolism, and mitochondrial metabolism converge at shared metabolic hubs, including acetyl-CoA, NADPH, *α*-ketoglutarate (*α*-KG), and ATP. These hubs integrate carbon, nitrogen, and redox metabolism to support biosynthesis, epigenetic remodeling, redox homeostasis, and energy producti on. Through this integrated network, cancer cells may dynamically adapt to metabolic stress and therapeutic interventions, leading to phenotypes such as metabolic plasticity, immune evasion, survival under treatment, and ultimately drug resistance. This schematic highlights how cross-pathway metabolic integration, rather than single-pathway alterations, underlies therapy resistance in cancer. HIF-1*α*, hypoxia-inducible factor 1*α*; mTORC 1, mechanistic target of rapamycin complex 1; SREBP, sterol regulatory element binding proteins; AMPK, AMP- activated protein kinase; NRF 2, nuclear factor erythroid 2-related factor 2; PGC 1A, peroxisome proliferator-activated receptor gamma coactivator 1A; p53, tumor protein p53; FAO, fatty acid oxidation; PPP, pentose phosphate pathway; GLS 1, glutaminase type-1; OXPHOS, oxidative phosphorylation; GSH, glutathione; ROS, reactive oxygen species.

This intricate composition dictates an intense metabolic interplay within the TME, which serves as a central engine driving metabolic reprogramming and subsequent drug resistance (Lim, 2024). On one hand, the hypoxic and acidic features of the TME activate transcription factors such as HIF-1*α*, forcing cancer cells to shift from oxidative phosphorylation to highly efficient glycolysis and other compensatory pathways, thereby reducing the efficacy of mitochondrial-targeted drugs or conventional chemotherapies (Liu et al., 2024). On the other hand, the metabolic crosstalk between cancer cells and TME components (such as CAFs and immune cells) establishes a sophisticated “metabolic defense system”. Cancer cells competitively deprive the niche of glucose and amino acids, plunging immune cells into a state of “metabolic starvation” to evade immune surveillance (Zhang et al., 2023). Furthermore, cellular senescence within the TME aggravates this environment *via* the senescence-associated secretory phenotype (SASP), releasing inflammatory factors that promote metabolic plasticity and immunosuppression (Zhang et al., 2024a). When clinical interventions block a primary metabolic pathway, the TME can facilitate stromal support mechanisms to release alternative nutrient sources or induce a state of metabolic dormancy in cancer cells to bypass the therapeutic strike (Dzobo, 2020; Zhang et al., 2024a). This high degree of metabolic plasticity and symbiosis allows tumors to adjust their energy acquisition in real-time, contributing to therapeutic resistance. Figure 2 illustrates the cross-pathway integration of metabolic reprogramming in the tumor microenvironment. The following sections will discuss these specific reprogrammed pathways in detail.

### Reprogrammed pathways

Metabolic reprogramming is a significant factor of drug resistance. A comprehensive understanding of these mechanisms is critical for developing precision strategies to diminish resistance. In this section, the identified reprogrammed pathway mechanisms will be discussed with reviews of recent research updates, followed by corresponding strategies for those drug targets. We have summarized the mechanisms underlying metabolic reprogramming in the following Table 1.

### Warburg effect

The Warburg effect, also known as aerobic glycolysis and first described by Otto Warburg in 1924, is a hallmark of cancer metabolism wherein tumor cells preferentially metabolize glucose *via* glycolysis. Instead of relying on mitochondrial oxidative phosphorylation, cancer cells produce lactate even under oxygen-rich conditions (Warburg, Wind & Negelein, 1927).

This metabolic reprogramming is driven by the overexpression of key glycolytic transporters and enzymes, including glucose transporters like glucose transporter (GLUT), hexokinase 2 (HK2), phosphofructokinase (PFK), pyruvate kinase M2 (PKM2), lactate dehydrogenase A (LDHA), and monocarboxylate transporters (MCT1, MCT4) (Fukushi et al., 2022; Yadav et al., 2024). Different GLUT subtypes, characterized by high glucose affinity, are differentially expressed across cancer cells, which often overexpress GLUT to meet elevated glucose uptake demands. Cytoplasm-localized HKs catalyze glucose phosphorylation, the first rate-limiting step of glycolysis. The HK2 subtype translocates to the outer mitochondrial membrane, where it interacts with voltage-dependent anion channels to confer chemoresistance under adverse conditions like hypoxia or chemotherapy, HK1 sustains glycolytic energy supply while HK2 suppresses apoptotic factor release. In the cytoplasm, cytoplasmic PFK overexpression boosts glycolysis and drives drug resistance. PFKFB3, upregulated by signals like hypoxia, estrogen receptor activation, RAS activation, and P53 deletion in cancer, enhances kinase activity to augment glycolytic flux. In liver cancer, it induces sorafenib resistance through the PFKFB3/HIF-1A positive feedback loop, while its inhibition restores oxaliplatin cytotoxicity in colorectal cancer by suppressing defensive autophagy. However, PFKFB3 inhibitors face limitations: cisplatin-induced acetylation (K472) impairs nuclear localization, but cytoplasmic accumulation still promotes glycolysis to counteract cisplatin; combining antiangiogenic therapy with PFKFB3 inhibition may disrupt endothelial metabolism or cause vascular leakage, hindering drug delivery. Collectively, these molecules enhance glucose uptake, glycolytic flux, and lactate efflux. MYC, as a transcription factor, directly induces GLUT, HK2, and PKM2 expression, and C-MYC synergizes with HIF1 to drive glycolysis, fostering tumor microenvironment (TME) formation and chemoresistance. Despite extensive mechanistic evidence linking enhanced glycolysis to drug resistance, it is important to note that much of this evidence is derived from *in vitro* studies or xenograft models. These systems often rely on supraphysiological glucose concentrations, which may overestimate the dependence of tumor cells on aerobic glycolysis compared with *in vivo* conditions. Moreover, genetic or pharmacological inhibition of glycolytic enzymes does not uniformly sensitize tumors to therapy, underscoring the context-dependent contribution of the Warburg effect to resistance. The Warburg effect thus reflects the integrated interplay of metabolic rewiring, oncogenic signaling, and TME adaptation, context-dependent metabolic feature that may contribute to drug resistance, although its therapeutic exploitability remains under active investigation (Liu, Jin & Fan, 2021a). Although oxygen-rich environment causes HIF-1*α* to be deactivated and destroyed by the ubiquitin proteasome pathway (UPP), oxygen deficiency enables HIF-1*α* to escape destruction and upregulate many genes involved in cancer progression (Rashid et al., 2021). Thus, the Warburg effect represents an integrated interplay of metabolic rewiring, oncogenic signaling, and TME adaptation. Studies on it unveil tumor survival, providing new perspectives for novel therapeutic strategies targeting this metabolic vulnerability, making it a critical target for overcoming drug resistance in cancer.

**Table 1:** Mechanisms of metabolic reprogramming.

Category	Core metabolic alteration	Representative drug resistance	Key limitations	Reference
Warburg effect	Enhanced aerobic glycolysis driven by upregulation of glucose transporters (GLUTs) and glycolytic enzymes (HK2, LDHA), regulated by oncogenic signaling and hypoxia-responsive pathways (HIF-1*α*, MYC, PI3K-AKT-mTOR)	GLUT3 causes TMZ resistance; HK2 inhibits apoptosis; PFKFB3 leads to sorafenib/cisplatin resistance	Effects are highly tumor-type and context dependent; glycolysis inhibition often triggers compensatory metabolic pathways, limiting durable responses.	Chen et al. (2007); Gogvadze, Zhivotovsky & Orrenius (2010); Liu, Jin & Fan (2021a); Rashid et al. (2021)
Lactate metabolism	Increased lactate production and export mediated by HIF-1*α*/c-MYC signaling and monocarboxylate transporters (MCT4/MCT1); lactate also acts as a signaling metabolite *via* histone lactylation	Lactate causes bevacizumab/chemotherapy/ anti-PD-1 ICI resistance in CRC/OS/melanoma	Lactate effects vary by cellular context and tumor compartment; some immune and stromal cells utilize lactate as a metabolic substrate, complicating therapeutic targeting	Chen et al. (2021); Li et al. (2023); Li et al. (2024b); Wang et al. (2024a); Liu et al. (2024)
Glutamine addiction	Dependence on glutamine to sustain biosynthesis, redox balance, and anaplerosis, driven by oncogenic regulators (c-MYC, KRAS, HIF) that upregulate GLS1 and ASCT2	Glutamine causes CB-839/CDK4/6 inhibitor resistance; ammonia induces autophagy	Metabolic plasticity allows adaptation *via* alternative anaplerotic substrates or nutrient sources; responses to glutamine-targeted therapies are heterogeneous	Jin et al. (2020); Li et al. (2024a); Qie et al. (2019); Ren et al. (2023); Vanhove et al. (2019)
Lipid synthesis rewiring	SREBPs regulate FASN for de novo lipogenesis; fatty acid oxidation enhanced; DGAT stores PUFAs to reduce peroxidation	FASN supports resistance; PUFAs reduce 5-FU sensitivity; lipid uptake offsets FASN inhibition	Lipid uptake from the microenvironment can bypass endogenous synthesis inhibition; lipid metabolism varies widely across tumor types	Cheng et al. (2024); Lee et al. (2024); Tiwari et al. (2025); Vanauberg, Schulz & Lefebvre (2023)
Mitochondrial reprogramming	Retention of oxidative phosphorylation, accumulation of oncometabolites from TCA cycle enzyme mutations, and altered mitochondrial dynamics and Ca^2^^+^ handling	NRF2 causes BRAF inhibitor resistance; 2-HG silences tumor suppressors; BCL-2 resists apoptosis	Tumors display heterogeneous reliance on mitochondrial metabolism; inhibition may preferentially affect subsets of cancer cells	Annesley & Fisher (2019); Huang, Radi & Arbiser (2021); Jin et al. (2022); Luo, Ma & Lu (2020); Mamouni, Kallifatidis & Lokeshwar (2021); Vasan, Werner & Chandel (2020)
Ion reprogramming	Dysregulation of Na^+^, K^+^, and Ca^2+^ channels (VGSCs, Kv channels, MCU) affecting membrane potential, metabolic flux, and mitochondrial function.	High Na^+^ reduces drug uptake; K^+^ offsets inhibitors; abnormal Ca^2+^ reduces TG sensitivity	Evidence remains largely correlative and tumor-specific; causal links to resistance require further validation	Brackenbury & Palmieri (2023); Bruce & James (2020); Huang & Jan (2014); James et al. (2022); Ratanasrimetha, Workeneh & Seethapathy (2022); Wawrzkiewicz-Jałowiecka et al. (2023)

The Warburg effect differs distinctly in various cancer types. For example, in colorectal cancer (CRC), the Warburg effect is driven by the overexpresion of glucose transporters and key glycolytic enzymes, which enhance glycolytic flux and lactate production, even under oxygen-rich conditions. This metabolic shift supports rapid ATP generation and biosynthesis precursor accumulation, collectively aggravating tumor proliferation and chemoresistance. Furthermore, to bypass glycolytic rate-limiting steps, CRC cells exploit fructose metabolism, and thus enhances redox balance through NADPH generation and stabilizes hypoxia-inducible factors, all of them play a vital role in promoting tumor survival, immune evasion, and metastasis (Pang & Wu, 2025). Glioblastoma uniquely depends on GLUT3—predominantly expressed in the nervous system and the most efficient GLUT isoform. Its overexpression endows glioblastoma cells with survival advantages under glucose limitation and enhances invasiveness, capabilities not shared by GLUT1. Notably, GLUT3 drives temozolomide (TMZ) resistance *via* the C-MYC/GLUT3 signaling axis. In this cancer, the Warburg effect fulfills the high energy requirements of rapid proliferation while interacting with PI3K/AKT/mTOR and HIF-1*α* pathways to reinforce tumor survival, metastasis, and therapeutic resistance, highlighting its critical role in progression and potential as a therapeutic target (Liu, Jin & Fan, 2021a). These cancer-type-specific differences highlight that the Warburg effect does not represent a uniform metabolic program across malignancies. Instead, its functional contribution to drug resistance is shaped by tissue origin, oncogenic drivers, and microenvironmental constraints. Such heterogeneity partially explains the inconsistent therapeutic responses observed when targeting glycolysis across different tumor models.

To tackle these challenges, a variety of experimental strategies have been explored, primarily in preclinical models, to target glycolytic enzymes or lactate transport. One approach focuses on direct inhibition of key glycolytic enzymes like oxamate for lactate dehydrogenase (LDH) by using small molecules to block their activity and impair tumor energy metabolism. After inhibiting LDH, the production of lactate from pyruvate is blocked, which can then be transported into mitochondrial for energy generation. Additionally, mitochondrial dysfunction, particularly mtDNA mutations, is addressed by restoring oxidative phosphorylation and suppressing glycolysis through p53 activation which induces the expression of TIGAR and SCO2, or correcting nuclear DNA defects in enzymes like succinate dehydrogenase (SDH) and fumarate hydratase (FH), which play important roles in tricarboxylic acid (TCA) cycle and mitochondrial complex II function. Moreover, there are also a nonclassical TCA cycle in nucleus that wires the metabolic-epigenetic circuitry (Liu et al., 2021b). Oncogenic signaling pathways such as PI3K/Akt/mTOR and Ras have also been targeted. 2-deoxyglucose with mTOR inhibitors achieves combined inhibition of both glycolysis and these pathways, demonstrating synergistic effects (Chen et al., 2007). The TME, particularly HIF-1*α* stabilization triggered by oxygen deficiency, is another critical focus. Suppression of HIF-1*α* downregulates its downstream glycolytic enzymes. This strategy is exemplified by the natural compound wogonin, which reverses hypoxia resistance through inhibition of the PI3K/Akt pathway. This breaks HIF-1*α* stabilization and subsequently reduces the expression of HK2, PDHK1 and LDHA, thereby diminishing glucose uptake and lactate production (Wang et al., 2013). Together, through disrupting glycolytic flux, restoring mitochondrial function, and modulating upstream regulators like HIF-1*α* and oncogenic kinases, these approaches demonstrate proof-of-concept evidence that perturbing glycolytic flux or its upstream regulators may modulate tumor sensitivity to therapy, although robust clinical validation is still lacking. The integration of these strategies highlights the potential for targeting metabolic vulnerabilities to enhance therapeutic efficacy in cancer treatment. Importantly, while these strategies provide valuable mechanistic insights and preclinical proof-of-concept, most interventions targeting the Warburg effect have not yet demonstrated consistent efficacy in clinical trials. This gap emphasizes the need for biomarker-guided patient stratification and rational combination strategies to translate metabolic targeting into clinical benefit.

However, despite the extensive evidence supporting enhanced aerobic glycolysis as a hallmark of cancer cells and its role in drug resistance, several limitations should be considered. Many studies linking the Warburg effect to therapeutic resistance are primarily based on established cancer cell lines cultured under high-glucose conditions, which may exaggerate glycolytic dependence compared with *in vivo* tumors. Importantly, the contribution of the Warburg effect to drug resistance appears to be highly context-dependent, varying across cancer types and treatment modalities. In some settings, glycolytic reprogramming may, in certain contexts, represent an adaptive metabolic consequence of therapy-induced stress rather than a primary causal driver of resistance.

### Lactate metabolism

**Figure 3:**
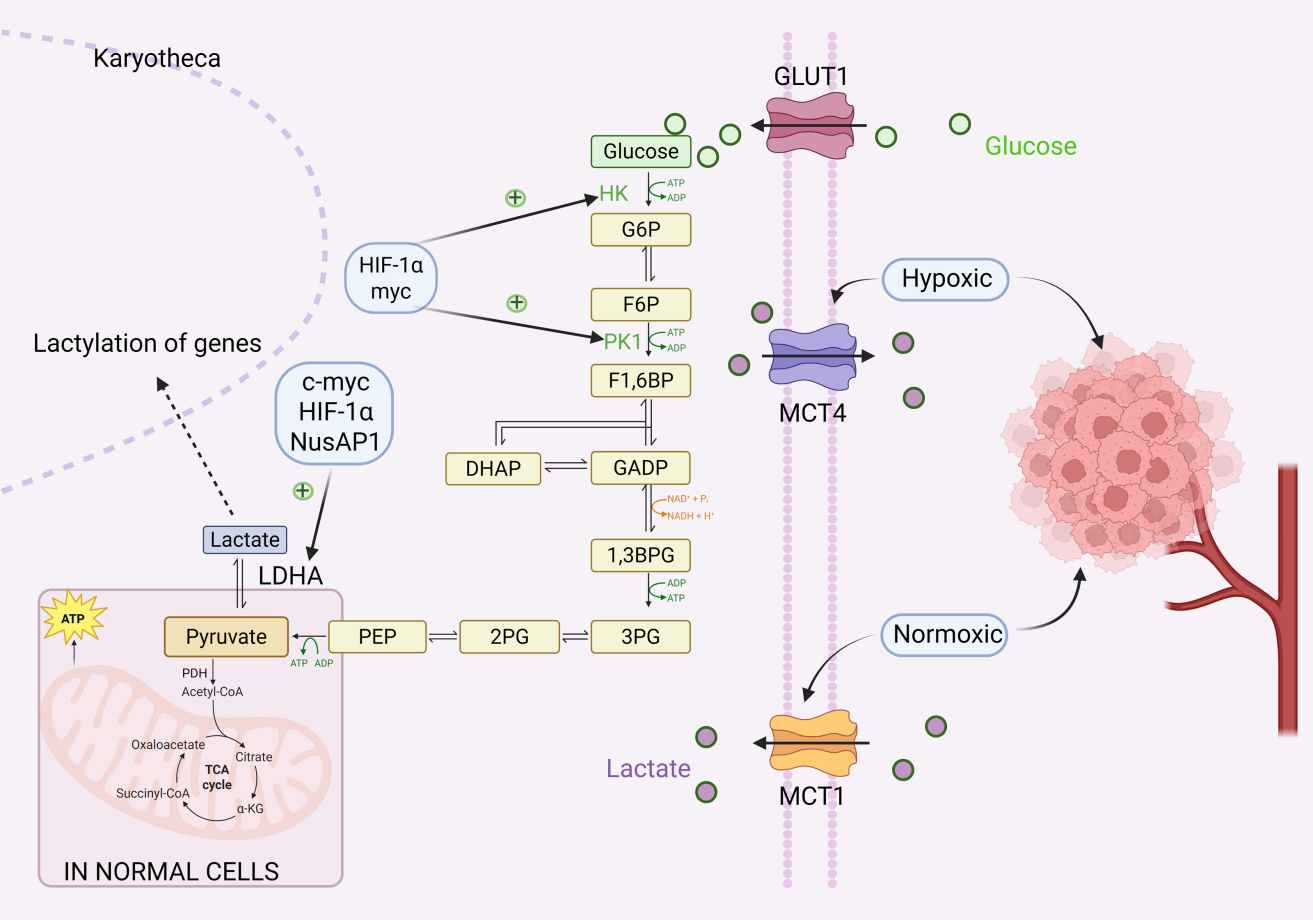
Lactate metabolism in cancer cells. Metabolic reprogramming in cancer cells compared to normal cells, emphasizing lactate metabolism and tumor microenvironment interactions. On the right, secreted lactate is transported across membranes, MCT4 for efflux from hypoxic cancer cells, and MCT1 for uptake by normoxic cells in nearby vessels or stromal cells. On the left, intracellular lactate serves as a substrate for post-translational modiûc ation lactylation. GLUT1, Glucose Transporter 1; HK, Hexokinase; G6P, Glucose-6-Phosphate; F6P, Fructose-6-Phosphate; F1,6BP, Fructose-1,6-Bisph osphate; DHAP, Dihydroxyacetone Phosphate; 1,3BPG, 1,3-Bisphosphoglycerate; 3PG, 3-Phosphoglycerate; 2PG, 2-Phosphoglycerate; PEP, Phosphoenolpyruvate; LDHA, Lactate Dehydrogenase A; HIF-1*α*, Hypoxia-inducible factor 1*α*; cMyc, Cellular Myc; MCT1, Monocarboxylate Transporter 1; MCT4, Monoca rboxylate Transporter 4.

Lactate metabolism is another core feature of cancer metabolic reprogramming, extending beyond mere byproduct generation, acting as an emerging metabolic signal implicated in cancer progression and epigenetic regulation. In cancer cells, lactate metabolism is characterized by enhanced production, selective transport, and functional utilization. Glycolytic flux is upregulated *via* overexpression of glucose transporters like GLUT1 and rate-limiting enzymes like hexokinase 2 (HK2) and lactate dehydrogenase A (LDHA), driving pyruvate toward lactate conversion rather than oxidative phosphorylation. This process is coordinated by hypoxia-responsive and oncogenic signaling, with hypoxia-inducible factor-1*α* (HIF-1*α*) serving as a central regulator. It binds to hypoxia-responsive elements (HRE) in the promoter regions aforementioned genes, boosting lactate synthesis. Oncogene c-Myc modulates glycolytic and glutaminolysis pathways to further increase lactate production. Secreted lactate is transported across membranes *via* monocarboxylate transporters (MCT), MCT4 for efflux from hypoxic cancer cells, and MCT1 for uptake by normoxic cells in nearby vessels or stromal cells. These mechanisms are schematically illustrated in Fig. 3. While intracellular lactate serves as a substrate for lysine lactylation (Kla)-a post-translational modification. Histone lactylation has been proposed as a mechanistic link between metabolic reprogramming and immune modulation (Cai et al., 2025). H3K18lahas been found to induce the transition from pro-inflammatory M1 to anti-inflammatory M2 macrophages, and non-histone protein function, thereby serving as an important bridge between metabolic reprogramming and epigenetics regulating gene transcription related to cancer cell proliferation and immune evasion (Chen et al., 2021; Li et al., 2024b; Wang et al., 2024a). Despite strong mechanistic support for lactate as a signaling metabolite, most evidence derives from *in vitro* systems or murine models. Genetic or pharmacological perturbation of lactate production or transport does not uniformly sensitize tumors to therapy, suggesting that lactate metabolism functions as a modulatory, rather than universal, driver of resistance.

Different cancers exhibit distinct lactate metabolic patterns. In osteosarcoma (OS), single-cell RNA sequencing identifies a lactate-high subcluster (OS cluster 2) with elevated glycolytic gene expression. Ubiquinone Oxidoreductase Complex Assembly Factor 6 (NDUFAF6), a hub lactate metabolism-related gene (LRG), promotes OS cell viability and invasion by regulating mitochondrial function and mediating MIF-driven metabolic communication (Wang et al., 2024a). In colorectal cancer (CRC), lactate-induced histone H3 lysine 18 lactylation (H3K18la) enriches at the promoter region of autophagy enhancer protein RUBCNL, activating its transcription. RUBCNL then interacts with BECN1 to recruit the class III phosphatidylinositol 3-kinase complex, facilitating autophagosome maturation and supporting CRC cell survival (Li et al., 2023). In triple-negative breast cancer (TNBC), tumor cells far from blood vessels are under hypoxic conditions, those cells secrete lactate *via* monocarboxylate transporter 4 (MCT4), while normoxic tumor cells, those near blood vessels, or cancer-associated fibroblasts (CAFs) take up lactate *via* MCT1 to fuel oxidative phosphorylation, forming a metabolic symbiosis that sustains tumor growth (Chen et al., 2022). These findings underscore that lactate metabolism is highly tumor-type-specific and context-dependent, limiting the generalizability of individual mechanistic observations.

Lactate-associated metabolic and epigenetic changes have been implicated in drug resistance. In CRC, high lactate levels increase H3K18la and RUBCNL expression, enhancing autophagy to help cancer cells survive bevacizumab-induced hypoxia, leading to resistance to this anti-angiogenic therapy (Li et al., 2023). In OS, elevated NDUFAF6 expression correlates with increased monocyte infiltration and reduced sensitivity to chemotherapy, as NDUFAF6-dependent lactate metabolism supports OS cell adaptation to therapeutic stress (Wang et al., 2024a). In melanoma, LDHA-mediated lactate accumulation suppresses the cytotoxic function of T cells by inhibiting the phosphorylation of p38 signaling protein and induces T-cell apoptosis by reducing the levels of nicotinamide adenine dinucleotide (NAD^+^). High lactate level also affects natural killer (NK) cell cytotoxic activity by reducing intracellular pH and inducing apoptosis of NK cells. All of above combined blunt the efficacy of anti-PD-1 immune checkpoint inhibitors (ICIs) (Chen et al., 2022). Importantly, lactate exerts differential effects on tumor cells and immune populations. While cancer cells may utilize lactate as a metabolic fuel or epigenetic signal, immune cells, particularly cytotoxic T cells and NK cells, appear more vulnerable to lactate-induced metabolic stress. This asymmetry further complicates therapeutic targeting of lactate metabolism.

Several experimental therapeutic strategies targeting lactate metabolism have been explored, primarily in preclinical models. For example, LDHA inhibitor GSK2837808A reduces lactate production and restores T-cell function in melanoma models (Chen et al., 2022). Combination of inhibition of histone lactylation by oxamate, inhibition of autophagy by chloroquine, and inhibition of immune checkpoint by anti-PD-1, significantly improves therapeutic efficacy in CRC patient-derived xenografts (PDXs) and organoids (PDOs), and enhances the sensitivity of CRC cells to bevacizumab treatment (Li et al., 2023). However, despite encouraging preclinical results, no lactate-targeting strategy has yet demonstrated consistent clinical benefit. These findings highlight the need for spatially resolved metabolic profiling and patient stratification to identify contexts in which lactate-directed therapies may be effective.

While accumulating evidence implicates lactate metabolism and transport in therapy resistance, the strength and causality of these associations vary substantially across experimental systems. Many studies emphasize the role that lactate plays in promoting tumor progression just based on the correlative associations between lactate levels, transporter expression, and resistance phenotypes. However, lactate can exert diverse and sometimes opposing effects depending on cellular context, tumor compartment, and metabolic state. Furthermore, experimental systems often do not fully recapitulate the spatial heterogeneity of lactate gradients observed in human tumors. These factors complicate efforts to establish a unified model linking lactate metabolism directly to drug resistance.

### Glutamine addiction

While Warburg effect is notorious for its role in cancer progression and drug resistance, amino acid metabolism also plays a pivotal role. Glutamine addiction exemplifies this metabolic reprogramming, referring to a context-dependent metabolic state in which certain cancer cells exhibit increased reliance on exogenous glutamine to fuel anabolic processes and maintain redox homeostasis due to aberrantly activated oncogenes and the loss of tumor suppressors (Choi & Park, 2018). This dependency arises from glutamine’s dual role as a nitrogen donor for biosynthesis of purine and pyrimidine nucleotides and a carbon source for anaplerosis, providing various intermediates in TCA cycle and for amino acid and lipid synthesis. Glutamine can influence gene expression *via* epigenetic mechanisms, which is emaplified in V600EBRAF melanoma cells by histone hypermethylation, leading to resistance to BRAF inhibitor treatment (Pan et al., 2016). Specifically, mechanistic target of rapamycin complex 1 (mTORC1), a central regulator of cell growth and anabolic metabolism, is activated by intracellular glutamine pools. Furthermore, efflux of intracellular glutamine also play a role *via* SLC7A5, which can drive leucine uptake, another amino acid that is required for mTORC1 activation (Nicklin et al., 2009). Oncogenic drivers such as C-MYC, KRAS, and HIF amplify glutamine uptake and catabolism by upregulating relating enzymes and transporters like glutaminase type-1 (GLS1) and alanine-serine-cysteine transporter, type-2 (ASCT2, encoded by gene SLC1A5), creating a putative metabolic vulnerability in a subset of malignancies (Li et al., 2024a). Notably, most mechanistic insights into glutamine addiction are derived from *in vitro* cultures or genetically engineered mouse models. These systems often fail to capture the dynamic nutrient competition and metabolic flexibility present in human tumors, potentially overestimating glutamine dependence.

The molecular mechanisms of glutamine addiction vary across cancer types, reflecting tissue-specific metabolic reprogramming. While noncompetitive allosteric GLS1 inhibitor CB-839 shows significant anti-proliferative activity in many types of cancer cell like lymphoma canciner and triple-negative breast cancer, it exhibits limited efficacy in hepatocellular carcinoma cells (HCC) due to compensatory metabolic plasticity. However, using V-9302 for glutamine transporter ASCT2 inhibition can sensitizing glutamine dependent (GD) cells to CB-839 treatment, thus achieving dual targeting of glutaminolysis and transport, which demonstrates enhanced efficacy in preclinical models. The combination of CB-839 and the ASCT2 inhibitor V-9302 sharply reduces glutathione and induces lethal oxidative stress (Jin et al., 2020). Similarly, esophageal squamous cell carcinoma (ESCC) with Fbxo4-cyclin D1 axis dysregulation exhibits mTORC1-driven glutamine dependence, sensitizing tumors to GLS1 inhibition paired with mitochondrial disruptors like metformin. This strategy also conquers the acquired resistance to CDK4/6 inhibitors (Qie et al., 2019). Together, these findings underscore that glutamine dependency varies markedly across tumor types and oncogenic contexts. Even within glutamine-dependent tumors, adaptive metabolic rewiring frequently limits the durability of glutaminase-targeted therapies.

Pancreatic and lung cancers further illustrate the tissue-specific regulation of glutamine metabolism. Kras mutations are a common type in pancreatic cancer. While mutant pancreatic tumors rely on glutamine-derived *α*-ketoglutarate to sustain nucleotide and lipid biosynthesis under nutrient-deficient conditions, lung cancer with c-Myc oncogene amplification upregulate GLS1 to support redox balance *via* glutathione synthesis (Ren et al., 2023; Vanhove et al., 2019). These adaptations create metabolic bottlenecks that may be exploitable in carefully defined contexts, but also foster drug resistance.

Recent studies have explored the therapeutic potential of targeting glutamine metabolic pathways in cancer cells, primarily in preclinical settings. Therapeutic resistance emerges *via* multiple mechanisms, including metabolic rewiring and antioxidant adaptation. Importantly, many resistance-associated metabolic adaptations emerge as compensatory responses to glutamine blockade rather than reflecting an intrinsic and fixed dependency on glutamine metabolism. Cancer cells compensate for glutamine blockade by activating alternative nutrient pathways, such as glycolysis or fatty acid oxidation, while upregulating glutathione synthesis mediated by nuclear factor erythroid 2-related factor 2 (NRF2), which in turn induce SLC7A11 expression to promote glutamine excretion in exchange for an increase in intracellular cysteine and thus alleviate oxidative damage (Li et al., 2024a; Obara-Michlewska & Szeliga, 2020). Glutamine-derived GSH can neutralize reactive oxygen species (ROS) produced during chemotherapy or radiation. At the same time, ammonia, a catabolite of glutamine, can induce autophagy to promote cancer cell survival. Oncogenic drivers like c-Myc and Kras increase glutamine uptake through SLC1A5 transporters, creating metabolic redundancy that helps tumors resist targeted therapies. For instance, in ESCC cells with cyclin D1 overexpression, there is an mTORC1-dependent glutamine dependency, which allows these cells to bypass the effects of CDK4/6 inhibitors. To combat the challenges posed by glutamine addiction, various therapeutic strategies have emerged. GLS1 inhibitor like CB-839 can trigger Gln accumulation in liver tissues and tumor xenografts. Combined treatment with CB-839 and metformin has shown proof-of-concept efficacy in preclinical models of tumors resistant to CDK4/6 inhibitors (Jin et al., 2020; Qie et al., 2019). While the concept of glutamine addiction is widely discussed, its universality and therapeutic relevance remain debated. Nevertheless, this dependency may not be universal. Several studies have shown that cancer cells can adapt to glutamine limitation by upregulating pyruvate carboxylation and activating alternative anaplerotic pathways (Cheng et al., 2011), or increasing reliance on other nutrients like (Vander Heiden & De Berardinis, 2017). In addition, pharmacological inhibition of glutamine metabolism often produces heterogeneous responses, like GLS inhibitor mentioned before (Jin et al., 2020), reflecting metabolic plasticity rather than a fixed vulnerability. These observations suggest that glutamine addiction represents a conditional phenotype shaped by oncogenic context, nutrient availability, and therapeutic pressure.

Collectively, these observations indicate that glutamine addiction represents a conditional metabolic phenotype shaped by oncogenic drivers, nutrient availability, and therapeutic pressure, rather than a universal metabolic vulnerability.

### Lipid synthesis rewiring

As another vital pathway of metabolic reprogramming, lipid metabolism is characterized by a significant dependence on exogenous FAs, enhanced *de novo* lipogenesis (DNL), increased fatty acid oxidation (FAO), and altered lipid storage mechanisms, all of which have been implicated in tumor progression and adaptive survival of cancer cells in the TME. Lipid transporters, including cluster of differentiation 36 (CD36), fatty acid transport proteins (FATPs), and plasma membrane fatty acid-binding protein (FABPpm), facilitate the uptake of extracellular fatty acids (FAs) into cells (Koundouros & Poulogiannis, 2019). In cancer cells, such as hepatocellular carcinoma (HCC), enhanced fatty acid uptake is often driven by the upregulation of lipid transporters like CD36. This upregulation not only increases extracellular FA internalization but also activates alternative signaling pathways, including modulation of the ER-associated Reticulons (RTN) family gene (Nogo-B), and the CCAAT/enhancer-binding protein *β* (CEBP *β*) expression. Specifically, CD36-mediated uptake of oxidized low-density lipoprotein (oxLDL) induces CEBP *β* expression, which in turn directly upregulates Nogo-B. Nogo-B interacts with ATG5 to promote lipophagy, resulting in lysophosphatidic acid-enhanced YAP oncogenic activity. Thus, targeting the Nogo-B pathway shows great promise has shown mechanistic relevance in preclinical models (Tian et al., 2019). The upregulation of DNL, another key feature of this reprogramming, is partly related to the Warburg effect and to the overexpression of several metabolic enzymes like the upregulation of transcription factors sterol regulatory element binding proteins (SREBPs), which is one of the factors that lead to the overexpression of fatty acid synthase (FASN). FASN is pivotal for the increased production of fatty acids, and also promotes cell proliferation, cell invasion, metastasis and angiogenesis by enabling the building of lipid rafts and consequently to the localization of oncogenic receptors such as HER2 and c-Met in membrane microdomains. By consuming NADPH, H^+^ to synthesize palmitate, FASN reduces the levels of this co-substrate, thereby alleviating its allosteric inhibition on 6-phosphogluconate dehydrogenase (PGDH), the second enzyme in the oxidative branch of the pentose-phosphate pathway. This enhances PGDH activity, leading to increased production of ribulose-5-phosphate, which is subsequently converted to ribose-5-phosphate, a key precursor for DNA/RNA synthesis. Additionally, FASN stimulates protein synthesis by activating the mTOR in HepG2 and HCT116, further supporting cell proliferation. However, the relative contribution of these mechanisms appears to vary across tumor types and metabolic states. This lipogenic shift is reinforced by oncogenic signaling pathways such as PI3K/AKT/mTOR, which stabilize mature SREBPs post-translationally and enhance their transcriptional activity (Vanauberg, Schulz & Lefebvre, 2023). In hepatocellular carcinoma (HCC), the activation of SREBP1 has been strongly associated with increased tumor proliferation rate and poor prognosis. What’s more, SREBP1 activation is further enhanced by ammonia from glutamine catabolism, creating a link between amino acid and lipid metabolism to fuel tumor growth (Cheng et al., 2024). Notably, much of the evidence linking altered lipid uptake and *de novo* lipogenesis to drug resistance is derived from transcriptomic or protein expression analyses. Functional validation at the level of lipid flux, composition, and subcellular distribution remains limited in many studies.

Simultaneously, in nutrient-deprived regions, cancer cells enhance fatty acid oxidation (FAO) to meet their high energy demands, particularly under metabolic stress. This process is mediated by a series of proteins, one of them is carnitine palmitoyltransferase (CPT). FAO begins with the prior activation of FAs to fatty acyl-CoA esters. Then, carnitine palmitoyl transferase 1 (CPT1), which is located on the outer mitochondrial membrane, catalyzes the conversion of these fatty acyl-CoA esters to acyl-carnitine derivatives, facilitating their import into the mitochondrial matrix *via* carnitine-acylcarnitine translocase. After that, CPT2, on the inner membrane, cleaves the carnitine molecule and releases the fatty acid. The peroxisome proliferator-activated receptor (PPAR)-*γ* pathway plays a pivotal role in upregulating FAO and enhancing FA absorption by activating lipid transporters CD36. In nutrient-deprived or hypoxic tumor regions, cancer cells rely on FAO to generate ATP and NADH, supporting survival under adverse conditions (Cheng et al., 2024).

Lipid storage mechanisms further contribute to cancer cell adaptability, particularly in resisting ferroptosis, a form of cell death driven by upregulated lipid peroxidation at cellular membranes. Cancer cells sequester excess polyunsaturated fatty acids (PUFAs) into lipid droplets *via* diacylglycerol acyltransferase (DGAT)-mediated triacylglycerol (TAG) synthesis. This process especially stands out in slow-cycling, antimitotic cancer cells, where cell cycle arrest triggers DGAT-dependent lipid droplet formation. By storing PUFAs as TAGs, cancer cells reduce their incorporation into phospholipids, thereby minimizing membrane lipid peroxidation and ferroptosis susceptibility. This mechanism is exploited by certain chemoresistant tumor populations, particularly under prolonged therapeutic stress, such as 5-fluorouracil-resistant cancers, to escape cell death (Lee et al., 2024). Importantly, lipid oxidation and storage do not uniformly promote resistance. In some tumor contexts, enhanced FAO supports survival under stress, whereas in others, excessive lipid oxidation or impaired lipid storage may sensitize cells to ferroptosis or metabolic collapse.

Targeting the mechanisms in lipid metabolism for overcoming resistance is also promising. SREBP, one of the factors that lead to the overexpression of fatty acid synthase, is a potential target. Fatostatin is one of SREBP inhibitors which impedes SREBP-1 translocation from the ER to Golgi apparatus by lipid accumulation, especially polyunsaturated fatty acids, thereby inducing ER stress (Lee et al., 2024). Combinatorial approaches that target both lipid synthesis and immune checkpoints are also currently under clinical evaluation to leverage metabolic-immune crosstalk (Tiwari et al., 2025). Despite encouraging preclinical findings, the clinical translation of lipid-targeting strategies remains challenging due to metabolic redundancy, systemic lipid homeostasis, and potential toxicity in normal tissues. These limitations highlight the need for context-specific targeting and combination strategies. Although altered lipid synthesis and remodeling have been proved associated with resistance to multiple anticancer therapies, unresolved issues remain. Some mainly focus on changes in the expression of key lipogenic enzymes, but lack directly assessing lipid flux or composition at a functional level (Koundouros & Poulogiannis, 2019). Conflicting results have also been reported regarding whether increased lipid synthesis promotes resistance or reflects compensatory adaptation following treatment-induced stress (Vanauberg, Schulz & Lefebvre, 2023; Lee et al., 2024). These inconsistencies underscore the need for more integrative approaches to dissect lipid metabolism–drug resistance relationships.

### Mitochondrial metabolic reprogramming

Mitochondria, as an important organelle in the cell, play an indispensable role in energy production through oxidative phosphorylation (OXPHOS), regulation of metabolic intermediates *via* the tricarboxylic acid (TCA) cycle, and maintenance of cellular homeostasis through dynamic fusion-fission processes (Annesley & Fisher, 2019). However, the metabolic reprogramming of cancer cells may enable the upregulation of compensatory pathways in the mitochondrial, such as glycolysis, to support their survival when mitochondrial metabolism is inhibited.

While the Warburg effect emphasizes aerobic glycolytic dominance, many cancer cells retain or reactivate OXPHOS to make up for their increased metabolism demands. Physiologically, normal prostate epithelial cells exhibit a unique metabolic phenotype characterized by the accumulation of zinc *via* ZIP1 transporters. This high concentration of intracellular zinc inhibits mitochondrial aconitase, effectively truncating the TCA cycle to preserve citrate for secretion into the seminal fluid. However, malignant transformation disrupts this process; prostate cancer cells lose the ability to accumulate zinc, leading to the reactivation of aconitase. Consequently, rather than being secreted, citrate is oxidized within the mitochondria to support lipogenesis, marking a fundamental shift from a citrate-accumulating phenotype to a malignant, citrate-oxidizing state (Mamouni, Kallifatidis & Lokeshwar, 2021). In melanoma, BRAF-mutant cells exhibit metabolic diversity, displaying high oxidative phosphorylation activity, employing a respiratory metabolic strategy to meet heightened energy needs under glucose deprivation. This adaptation is mediated by PGC1A-driven mitochondrial elongation and increased respiratory gene expression, which compensates for glycolytic inhibition and has been associated with resistance to BRAF inhibitors in specific metabolic contexts (Huang, Radi & Arbiser, 2021). Importantly, OXPHOS engagement varies not only between cancer types but also within tumor subpopulations, reflecting metabolic heterogeneity rather than a uniform resistance mechanism. The TCA cycle is also influenced, leading to the generation of biosynthetic precursors and oncometabolites. Mutations in isocitrate dehydrogenase (IDH)-1/2 lead to abnormal 2-hydroxyglutarate (2-HG) accumulation from D-*α*-ketoglutarate (D-*α*-KG), which competitively inhibits *α*-ketoglutarate-dependent dioxygenases like 50 -methylcytosine hydroxylase TET2, inducing genome-wide hypermethylation and the silencing of tumor suppressors (Luo, Ma & Lu, 2020). IDH3A was established to promote HCC development through regulation MTA1 expression (Liu et al., 2020). Similarly, loss-of-function mutations in succinate dehydrogenase (SDH) or fumarate hydratase (FH) respectively cause succinate or fumarate buildup (Vasan, Werner & Chandel, 2020). Such metabolic reprogramming contributes to tumor adaptation within the TME and may indirectly influence responses to therapy. Most evidence linking oncometabolite accumulation to therapy resistance is derived from correlative epigenetic and transcriptional analyses, whereas direct causal validation in treatment settings remains limited. While mitochondrial fission and fusion participate in the maintaining cellular homeostasis, these mitochondrial adaptive processes are also are dynamically regulated in response to cellular stress and have been implicated in tumor progression and therapy adaptation, examplified by the phosphorylation or activation of several upstream kinases, such as AMPK, cyclin B1/Cdk1, ERK1, and DRP1 (Jin et al., 2022; Srinivasan et al., 2017). This process is often accompanied by downregulation of fusion proteins (MFN1/2 and OPA1), impairing mitochondrial quality control and increasing susceptibility to ROS-induced damage. Notably, mitochondrial fission and fusion can exert opposing effects on cell fate, with excessive fission promoting apoptosis in some contexts while supporting survival and resistance in others. However, certain cancers, such as leukemic cells, exploit fusion *via* MFN2 to maintain OXPHOS efficiency and evade apoptosis. Mitochondrial Ca^2+^ homeostasis is another critical axis; the mitochondrial calcium uniporter (MCU) mediates Ca^2+^ uptake to support ATP production, but dysregulation can trigger mPTP opening and apoptosis. Cancer cells counteract this by overexpressing anti-apoptotic BCL-2 family proteins to stabilize the outer mitochondrial membrane (Genovese et al., 2021). Crosstalk between these mechanisms also exits. For example, TCA cycle dysfunction like IDH mutations destabilizes mitochondrial dynamics and increase accumulation of 2-hydroxyglutarate which brings about methylation and other epigenetic modifi-cation (Srinivasan et al., 2017). Such interactions enable tumors to resist therapy, emphasizing the need for combined targeting strategies.

**Figure 4:**
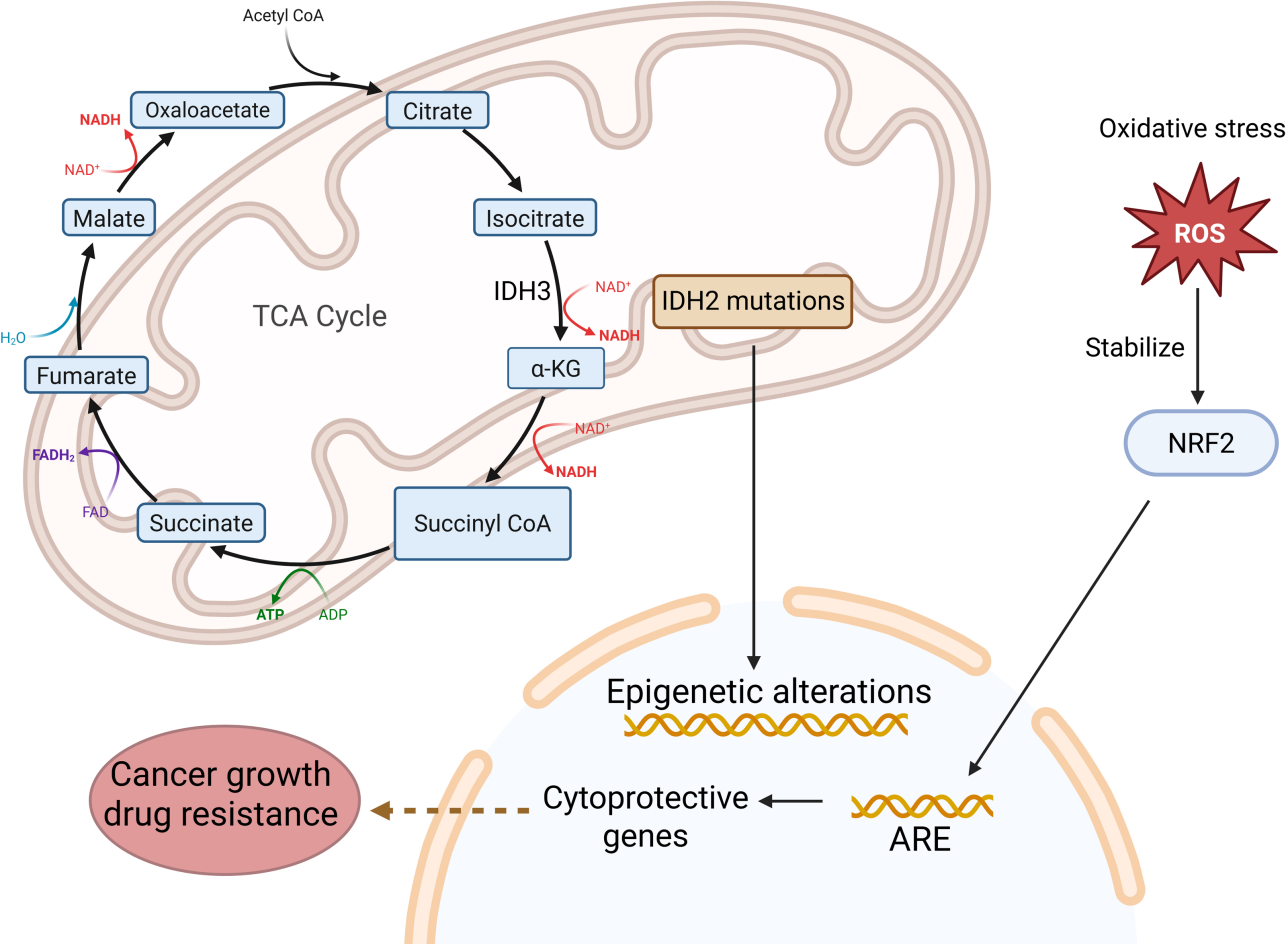
Mitochondrial reprogramming. Oxidative stress condition activates NRF2, which then is translocated to the nucleus where it binds to ARE, and triggers a considerable number of downstream cytoprotective genes like autophagy and apoptosis regulated genes, antioxidant and other enzymes. Mutations in IDH1 and IDH2 result in the production of oncometabolite D-2-hydroxyglutarate (D-2 HG) from *α*-KG. D-2 HG is an *α*-KG competitor, which means the elevated D-2 HG inhibits dioxygenase enzymes, leading to epigenetic landscape alterations through increasing histone and DNA methylation. NRF2, nuclear factor erythroid 2-related factor; IDH, isocitrate dehydrogenase; ARE, antioxidant response element.

The molecular mechanisms by which mitochondrial reprogramming confers drug resistance are complex. In BRAF inhibitor-resistant melanoma, resistance is achieved through the activation of the nuclear factor erythroid 2-related factor (NRF2) antioxidant pathway, which stabilizes mitochondrial function and reduces ROS-induced apoptosis (Baird & Yamamoto, 2023; Huang, Radi & Arbiser, 2021; Luo, Ma & Lu, 2020). Mutations in TCA cycle enzymes, such as IDH1/2, generate oncometabolites like 2-hydroxyglutarate (2-HG), which inhibit *α*-ketoglutarate-dependent dioxygenases, leading to DNA hypermethylation and epigenetic silencing of tumor suppressors (Luo, Ma & Lu, 2020; Xue, Zhou & Qiu, 2020) (Fig. 4). Additionally, mitochondrial Ca^2^^+^ overload, regulated by the mitochondrial calcium uniporter (MCU), can trigger the mitochondrial permeability transition pore (mPTP) opening, but cancer cells bypass this by overexpressing anti-apoptotic BCL-2 family proteins like MCL1 to stabilize the outer mitochondrial membrane (Genovese et al., 2021). Despite extensive preclinical exploration, targeting mitochondrial metabolism in cancer therapy is complicated by its essential role in normal tissues and the high degree of metabolic plasticity exhibited by tumor cells.

Targeting mitochondrial metabolism to overcome resistance requires precision strategies. The use of metformin to inhibit ETC complex I to block mitochondrial translation leads to the inhibition of OXPHOS, showing great promise in OXPHOS-dependent cancers, such as prostate cance (Mamouni, Kallifatidis & Lokeshwar, 2021; Vasan, Werner & Chandel, 2020). Disrupting mitochondrial dynamics with Drp1 inhibitors like Mdivi-1 or promoting fusion *via* MFN2 agonists can sensitize cells to apoptosis, particularly in cancers with hyperactive fission (Srinivasan et al., 2017). Integrated approaches, such as co-targeting glycolysis with inhibitor 2-deoxyglucose (2-DG) and OXPHOS with phenformin, exploit metabolic inflexibility to prevent compensatory adaptations (Jin et al., 2022). These strategies show the potential of mitochondrial metabolism as a therapeutic vulnerability.

Mitochondrial metabolic reprogramming has emerged as a crucial theme, however, the literature presents conflicting observations, with some studies reporting enhanced mitochondrial activity (Mamouni, Kallifatidis & Lokeshwar, 2021; Genovese et al., 2021), while others describe mitochondrial dysfunction or suppression (Genovese et al., 2021). These discrepancies likely reflect differences in cancer type, treatment history, and experimental systems. In addition, mitochondrial adaptations may represent reversible metabolic states rather than stable resistance mechanisms. Disentangling causality from association remains a major challenge in this area.

### Ion metabolic reprogramming

In cancer cells, ion metabolism also undergoes significant changes, for this part in the review, we specifically take about sodium (Na^+^), potassium (K^+^), and calcium (Ca^2^^+^) homeostasis, which collectively contribute to tumor progression and drug resistance. These changes include abnormal channel expression, disrupted electrochemical gradients, and dysregulated signaling pathways that sustain malignant phenotypes.

Elevated intracellular Na^+^ level, mediated by aberrant voltage-gated sodium channel (VGSC) activity, is a significant characteristic of aggressive breast cancer. These channels facilitate Na^+^ influx, which has been associated with activation of downstream signaling pathways linked to cell invasion and metastasis. This elevated Na^+^ accumulation is further linked to heightened cellularity and metastatic potential, as observed *via* ^23^Na MRI, where tumor Na^+^ correlates with malignancy and predicts treatment response (James et al., 2022). The abnormal Na^+^ dynamics also disrupt osmotic balance and cell volume regulation, further enhancing migratory potential. Notably, Na^+^ accumulation correlates with poor prognosis and has been implicated in reduced therapeutic responsiveness in some studies, as high Na^+^ may impair drug uptake or activate survival pathways (Ratanasrimetha, Workeneh & Seethapathy, 2022). Preclinical studies demonstrate that VGSC inhibitors like lidocaine can suppress metastasis, highlighting their potential as anti-metastatic agents (Brackenbury & Palmieri, 2023). However, although cariporide, inhibitor for Na^+^ dependent Na^+^/H^+^ exchanger (NHE1) which is related to tumor pH and metastasis, succeeds in reducing tumor growth and sensitizing cancer cells to chemotherapy, it also shows limited efficacy in reducing elevated Na^+^ concentration, calling for more specific interventions (James et al., 2022). Notably, most evidence linking sodium dysregulation to metastasis or therapy response is derived from imaging correlations and functional channel inhibition studies, rather than direct metabolic flux analyses.

Potassium channels, particularly voltage-gated (Kv) and calcium-activated (K_Ca_) families, are frequently overexpressed in cancers and have been reported to influence tumor proliferation, metabolic regulation, and immune cell function in a context-dependent manner. Kv1.3 channels regulate intracellular K^+^ levels, which influence hexokinase II activity, a key enzyme in glycolysis. Reduced K^+^ concentrations impair glycolysis, and K^+^ restoration reactivates ATP production. Similarly, mitochondrial potassium channels like mitoKv1.3 and mitoIK_Ca_ are overexpressed in cancers and are linked to reactive oxygen species (ROS) production and apoptosis resistance. Inhibition of these channels may cause ROS-mediated cell death, indicating their involvement in cancer cell survival. Additionally, Kv1.5 channels, downregulated in many cancers, are upregulated by dichloroacetate (DCA) in NFAT1-dependent way, promoting oxidative phosphorylation over glycolysis. Potassium channels also interact with metabolic enzymes such as pyruvate kinase (PK) and pyruvate dehydrogenase (PDH), which rely on K^+^ for activity. For example, PKM2, one of the isoforms of PK, is highly expressed in tumor cells. Its activity can be influenced by intracellular ionic conditions, including K^+^ availability, affecting the conversion of pyruvate to lactic acid. Furthermore, microRNAs like miR-125b-5p and miR-137 indirectly link potassium channels to metabolic reprogramming by targeting hexokinase and other glycolytic enzymes (Wawrzkiewicz-Jałowiecka et al., 2023). These molecular mechanisms show the intricate interplay between potassium channels and cancer metabolism, offering potential therapeutic targets. Importantly, potassium channels can exert both pro- and anti-tumorigenic effects depending on channel subtype, subcellular localization, and cellular metabolic state, complicating their therapeutic exploitation.

Calcium signaling is extensively reprogramed in cancers, with dysregulated Ca^2^^+^ channels, pumps, and binding proteins contributing to unchecked proliferation and apoptosis resistance. Mitochondrial Ca^2+^ uniporter (MCU) is responsible for Ca^2+^ uptake into the mitochondria. Mitochondrial Ca^2^^+^ overload leads to the production of reactive oxygen species (ROS), which then combined with Ca^2+^, activate the permeability transition pore (mPTP), mitochondrial membrane potential loss, ATP depletion and necrosis. Ca^2+^ overload can also activate calpain and cleavage of the PMCA, Na^+^/Ca^2+^-exchange (NCX), and inositol 1,4,5-trisphosphate receptors (IP_3_Rs) (Bruce & James, 2020). However, targeting calcium signaling poses substantial translational challenges, given the essential role of Ca^2^^+^ homeostasis in normal tissues and the narrow therapeutic window of many Ca^2^^+^-modulating agents. Pharmacological targeting of Ca^2^^+^ machinery, such as SERCA pump inhibitor, thapsigargin (TG), has demonstrated anti-tumor effects in preclinical models by inhibiting Ca^2+^ uptake into ER and deplete ER Ca^2+^ stores. But challenges remain due to non-selectivity. This could be conquered by using G202, in which an analogue to TG is conjugated to prostate specific membrane antigen (PSMA) targeting peptide. With high targeting and precision, G202 has successfully inhibited the proliferation of cancer cells in cancers such as bladder cancer and prostate cancer, with minimal toxicity (Cui et al., 2017).

The interplay between these ion fluxes creates a intricate network that amplifies oncogenic signaling. For example, K^+^ channel-mediated membrane potential changes modulate Ca^2^^+^ channel activity, creating feedback loops that sustain proliferation. Resistance to ion channel-targeted therapies often arises from compensatory overexpression of alternative channels or metabolic adaptations, necessitating combination approaches (Bruce & James, 2020; Cui et al., 2017). Overall, targeting ion metabolism offers a multifaceted strategy against cancer, but its clinical translation requires overcoming pharmacokinetic limitations and identifying predictive biomarkers for patient stratification. Unlike core metabolic pathways, ion homeostasis integrates biophysical regulation with signaling and metabolism, making it difficult to isolate direct causal relationships between ionic alterations and drug resistance.

Therapeutic strategies targeting ion reprogramming are emerging. VGSC blockers like lidocaine repress breast cancer metastasis by inhibiting Na^+^-dependent invasion pathways. Kv channel inhibitors like TRAM-34 disrupt metabolic adaptation and induce apoptosis (Brackenbury & Palmieri, 2023). Ca^2^^+^ channel modulators, such as SOCE inhibitors and TRPV1 antagonists like capsazepine, show promise in preclinical models by normalizing oncogenic Ca^2^^+^ signaling. Combining these strategies with conventional chemotherapy may overcome resistance, as ion channels often upregulate drug efflux pumps like P-glycoprotein (Cui et al., 2017). However, challenges remain due to the ubiquitous role of ions in normal physiology, necessitating tumor-specific delivery systems to minimize off-target effects. Compared with other metabolic pathways, ion metabolic reprogramming remains relatively underexplored in the context of drug resistance. Existing studies often focus on individual ions or transporters, such as calcium or iron, making it difficult to draw generalizable conclusions. Moreover, ion homeostasis is tightly interconnected with signaling pathways and cellular stress responses, complicating efforts to isolate direct metabolic effects. The lack of standardized experimental frameworks and limited clinical validation further highlight important gaps in the current literature.

### Additional metabolic pathways contributing to drug resistance

In addition to the major metabolic pathways discussed above, several auxiliary metabolic circuits have also been implicated in therapy resistance, including one-carbon metabolism, serine–glycine metabolism, and *de novo* purine and pyrimidine biosynthesis. These pathways support nucleotide production, methyl donor availability, and redox homeostasis, thereby enabling tumor cells to sustain rapid proliferation and adapt to therapeutic stress (Amelio et al., 2014; Ducker & Rabinowitz, 2017; Shi et al., 2023).

One-carbon metabolism, tightly linked to the serine–glycine pathway, provides one-carbon units required for folate and methionine cycles, which in turn fuel nucleotide biosynthesis and epigenetic methylation reactions that contribute to therapy tolerance and DNA damage repair. Enhanced serine–glycine flux has been shown to maintain NADPH production and antioxidant capacity, thereby buffering oxidative stress induced by chemotherapy or targeted therapy (Sun, Zhao & Cui, 2023).

Similarly, increased purine and pyrimidine biosynthesis represents a common adaptive mechanism in resistant tumors, ensuring continuous DNA and RNA synthesis and facilitating repair of therapy-induced DNA lesions. Upregulation of nucleotide metabolic enzymes, such as dihydroorotate dehydrogenase (DHODH) and ribonucleotide reductase, has been associated with resistance to multiple anticancer agents across different malignancies (Pranzini et al., 2022).

Importantly, these pathways are metabolically integrated with glycolysis, glutamine metabolism, and lipid synthesis through shared intermediates, including NADPH, acetyl-CoA, and *α*-ketoglutarate. Thus, rather than functioning as isolated metabolic modules, one-carbon metabolism, serine–glycine metabolism, and nucleotide biosynthesis act as interconnected auxiliary nodes that reinforce the core metabolic reprogramming network driving tumor survival and drug resistance (Pan et al., 2021).

### Clinical translation challenges of targeting metabolic reprogramming

Despite extensive mechanistic insights into metabolic reprogramming and its roles in drug resistance, translating these findings into effective clinical therapies remains challenging.

Tumor metabolic heterogeneity represents a major barrier to the clinical translation of metabolism-targeted therapies. Distinct metabolic phenotypes exist not only between different tumor types but also within different regions of the same tumor, driven by variations in microenvironmental conditions and genetic background. Such heterogeneity can lead to diverse responses to metabolic interventions and reduce the generalizability of strategies developed in preclinical models to patient cohorts. This phenomenon has been highlighted in reviews discussing the complexity of tumor metabolic landscapes and their implications for therapeutic targeting (Demicco et al., 2024; Tong, Gao & Liu, 2020).

Metabolic plasticity and compensatory pathway activation limit the effectiveness of monotherapies that target a single metabolic node. Cancer cells can rapidly rewire their metabolism in response to inhibition of a specific pathway, engaging alternative nutrient utilization routes to maintain energy production and survival. Such adaptive flexibility has been noted as a common challenge in efforts to exploit altered metabolism for therapy and complicates the identification of durable metabolic vulnerabilities *in vivo* (Alkaraki et al., 2021; Huang et al., 2025; Zhang et al., 2024b).

Moreover, the metabolic crosstalk between cancer cells and stromal cells within the tumor microenvironment (TME) further complicates translational success. Interactions between tumor cells, immune cells, fibroblasts, and endothelial cells create a metabolic network that can buffer metabolic stress imposed by pathway-specific inhibitors. Stromal and immune components can supply alternative nutrients or create compensatory metabolic niches, diminishing the efficacy of interventions designed to target tumor-intrinsic metabolic dependencies (Li et al., 2020; Zhang et al., 2025a).

In addition, the lack of robust clinical validation for many proposed metabolic strategies underscores a translational gap. Another critical yet underexplored barrier to clinical translation is the lack of validated metabolic biomarkers to guide patient stratification. Because metabolic dependencies differ markedly across tumor types and even within distinct tumor regions, therapies targeting specific metabolic pathways may only benefit metabolically defined subpopulations rather than unselected patient cohorts. For instance, elevated expression of glutaminase (GLS1) or the glutamine transporter SLC1A5 has been associated with glutamine-addicted tumors and may predict sensitivity to glutaminase inhibition in preclinical and translational studies (Jin et al., 2020; Li et al., 2024a). Similarly, increased intratumoral lactate accumulation and upregulation of monocarboxylate transporters (MCT1/MCT4) have been linked to immunosuppressive tumor microenvironments and reduced responsiveness to immune checkpoint blockade (Chen et al., 2022; Zhang et al., 2024b). Tumors characterized by enhanced oxidative phosphorylation (OXPHOS), often driven by PGC1*α*-dependent transcriptional programs, may display increased vulnerability to mitochondrial metabolism inhibitors, highlighting the importance of mitochondrial gene expression signatures as potential predictive biomarkers (Huang, Radi & Arbiser, 2021; Vasan, Werner & Chandel, 2020). In addition to molecular markers, metabolic imaging approaches such as 18F-fluorodeoxyglucose positron emission tomography (FDG-PET) or emerging hyperpolarized metabolic MRI provide non-invasive means to monitor tumor metabolic phenotypes and therapeutic responses *in vivo* (Tong, Gao & Liu, 2020; Vander Heiden, 2013). Collectively, integrating metabolic biomarkers into clinical trial design and therapeutic decision-making could substantially improve patient selection, enhance treatment efficacy, and bridge the gap between mechanistic metabolic insights and clinical application. A summary of representative predictive metabolic biomarkers and their clinical implications is provided in Table 2.

**Table 2:** Predictive metabolic biomarkers for patient stratification in metabolism-targeted therapies.

Metabolic dependency	Candidate biomarkers	Detection method	Clinical relevance	Key references
Glycolysis-dominant tumors	GLUT1, HK2, FDG uptake	IHC, PET imaging	Predict sensitivity to glycolysis inhibitors and aggressive phenotypes	Vander Heiden (2013) Tong, Gao & Liu (2020)
Lactate-driven immunosuppressive TME	Lactate level, MCT1/MCT4 expression	Metabolomics, IHC	Associated with immune evasion and reduced ICI response	Chen et al. (2022); Zhang et al. (2024a); Zhang et al. (2024b)
Glutamine addiction	GLS1, SLC1A5 (ASCT2) expression	RNA-seq, IHC	Identify tumors sensitive to glutaminase inhibition	Jin et al. (2020); Li et al. (2024a); Li et al. (2024b)
OXPHOS-dependent tumors	PGC1*α* signature, mitochondrial gene sets	Transcriptomics	Predict vulnerability to OXPHOS inhibitors	Huang, Radi & Arbiser (2021) Vasan, Werner & Chandel (2020)
Lipid metabolic rewiring	FASN, CD36 expression	IHC, lipidomics	Associated with lipogenesis-driven resistance and FA uptake reliance	Koundouros & Poulogiannis (2019); Cheng et al. (2024)

Although numerous metabolic targets and combinations have shown promise in preclinical models, including genetic perturbations, pharmacological inhibition, or combination approaches, relatively few have progressed to rigorous clinical testing with clear evidence of efficacy in patients. Challenges such as toxicity, precise patient selection, and measurable biomarkers for metabolic target engagement further complicate clinical development (Vander Heiden, 2013). Key reasons underlying the limited clinical success of metabolic inhibitors are summarized in Table 3.

**Table 3:** Clinical failure summary table.

Barrier	Mechanistic basis	Clinical consequence	Supporting evidence
Metabolic heterogeneity	Diverse metabolic phenotypes across tumors	Limited efficacy of metabolism-targeted therapies across cancer types (*e.g.*, IDH inhibitors effective in AML but not in solid tumor like glioma)	Luengo, Gui & Vander Heiden (2017)
Metabolic plasticity	Activation of alternative pathways	Rapid adaptive resistance	Boroughs & De Berardinis (2015)
TME metabolic crosstalk	Metabolic symbiosis and nutrient exchange between tumor and stromal cells	Reduced monotherapy efficacy	Li et al. (2020)
Systemic toxicity	Shared metabolism with normal tissues	Narrow therapeutic window	Vander Heiden (2013)
Limited predictive biomarkers for metabolic therapies	Heterogeneous metabolic dependencies across patients may dilute observable treatment effects in unselected clinical trials.	Heterogeneous metabolic dependencies across patients may dilute observable treatment effects in unselected clinical trials.	Luengo, Gui & Vander Heiden (2017); Vander Heiden & De Berardinis (2017)

In addition to these limitations observed with single-agent metabolic inhibitors, combination strategies targeting multiple metabolic pathways introduce further translational challenges. Although dual or multi-pathway metabolic inhibition has shown synergistic anti-tumor effects in preclinical studies, such approaches may exacerbate systemic toxicity because fundamental metabolic processes are also required for the function of normal proliferative tissues, including immune and gastrointestinal cells (Vander Heiden, 2013). Furthermore, co-targeting interconnected metabolic pathways can lead to complex intracellular metabolite perturbations, such as altered NADPH homeostasis, acetyl-CoA flux, and redox balance, which may influence both therapeutic efficacy and tolerability (Boroughs & De Berardinis, 2015; Pavlova & Thompson, 2016). Importantly, the optimal scheduling and dosing of combined metabolic inhibitors remain insufficiently defined, as simultaneous *versus* sequential inhibition of metabolic nodes can trigger distinct compensatory adaptations and reshape tumor metabolic plasticity *in vivo* (Vasan, Werner & Chandel, 2020). These considerations indicate that, although rational combination strategies hold promise for overcoming metabolic plasticity and resistance, their clinical implementation requires careful evaluation of additive toxicities, pathway crosstalk–driven metabolic interactions, and biomarker-guided patient selection. Altogether, these factors highlight that while metabolic reprogramming offers a compelling conceptual framework for therapeutic intervention, current strategies often remain at the preclinical or exploratory stage, with limited evidence of robust clinical benefit. Future efforts must integrate an understanding of tumor metabolic heterogeneity, compensatory adaptations, TME interactions, and translational validation to realize successful metabolism-based therapies. Importantly, beyond these conceptual barriers, several practical factors have contributed to the limited success of metabolic inhibitors in clinical trials. First, the systemic nature of core metabolic pathways often results in a narrow therapeutic window, as normal proliferative tissues such as immune cells and gastrointestinal epithelium share similar metabolic requirements with tumor cells, leading to dose-limiting toxicities that prevent achieving therapeutically effective concentrations in patients (Pavlova & Thompson, 2016; Vander Heiden, 2013). Second, metabolic dependencies identified in preclinical models are frequently context-dependent and may not be uniformly conserved across heterogeneous patient populations. Consequently, clinical trials conducted without biomarker-guided stratification may dilute potential therapeutic benefits that are restricted to metabolically defined tumor subgroups (Luengo, Gui & Vander Heiden, 2017). Third, tumors can undergo compensatory metabolic rewiring *in vivo*, activating parallel pathways such as fatty acid oxidation, serine–glycine metabolism, or enhanced mitochondrial respiration to bypass targeted metabolic inhibition. These adaptive responses reduce the durability and magnitude of clinical responses to single-agent metabolic therapies (Boroughs & De Berardinis, 2015; Pavlova & Thompson, 2016).

Collectively, these clinical observations indicate that the limited efficacy of metabolism-targeted therapies in trials does not necessarily negate their mechanistic rationale, but rather underscores the need for biomarker-driven patient selection, rational combination regimens, and dynamic metabolic monitoring to better translate metabolic vulnerabilities into durable clinical benefit (Vander Heiden, 2013).

## Discussion

In conclusion, metabolic reprogramming orchestrates drug resistance through interconnected pathways, each contributing to tumor survival under therapeutic pressure. The Warburg effect is also known as aerobic glycolysis where tumor cells preferentially metabolize glucose *via* glycolysis to produce lactate even under oxygen-rich conditions instead of relying on mitochondrial oxidative phosphorylation. Lactate metabolic reprogramming is characterized by enhanced lactate production driven by factors like HIF-1*α* and c-Myc, selective transport between cancer cells and stromal cells, and functional utilization such as mediating histone lactylation to regulate gene transcription related to cancer cell proliferation and immune evasion, and it contributes to cancer progression and drug resistance (Chen et al., 2021; Chen et al., 2022; Li et al., 2023; Li et al., 2024b; Wang et al., 2024a). Mitochondrial metabolic reprogramming involves altered functions such as oxidative phosphorylation, dynamics, and calcium homeostasis to support tumor adaptation and drug resistance (Annesley & Fisher, 2019; Luo, Ma & Lu, 2020; Mamouni, Kallifatidis & Lokeshwar, 2021; Vasan, Werner & Chandel, 2020). Amino acid metabolic reprogramming is characterized by glutamine addiction where cancer cells rely on exogenous glutamine for anabolic processes and redox homeostasis, and ion metabolic reprogramming includes dysregulated sodium, potassium, and calcium homeostasis with abnormal channel activity and signaling that contributes to tumor progression and drug resistance (Cui et al., 2017; Ratanasrimetha, Workeneh & Seethapathy, 2022; Vanhove et al., 2019). These mechanisms collectively enable tumors to combat therapeutic stress through metabolic redundancy and crosstalk with the TME.

Despite these advances, clinical translation of metabolic inhibitors faces significant challenges. Tumor heterogeneity, compensatory pathway activation, and stromal metabolic crosstalk often limit monotherapy efficacy. For example, GLS1 inhibitors like CB-839 show limited durability due to metabolic rewiring toward glycolysis or fatty acid oxidation (Qie et al., 2019). Stromal contributions, such as CAF-derived lactate in pancreatic cancer, further complicate therapeutic efficacy by creating a protective niche for residual disease (Mir et al., 2025; Zhu et al., 2022).

To overcome these barriers, future strategies may prioritize combined approaches to counteract therapy resistance driven by tumor metabolic reprogramming. Dual targeting of glycolysis and glutaminolysis like 2-DG with CB-839 or integrating lipid synthesis inhibitors with immunotherapy may enhance efficacy (Jin et al., 2020; Jin et al., 2022). To precisely guide such combination strategies, tools for real-time metabolic profiling are essential. Possible techniques include non-invasive sodium (^23^Na) MRI. Researches using ^23^Na MRI confirmed that the total sodium concentration ([Na^+^]) in tumor tissues was significantly higher than that in non-tumor tissues, and the abnormal elevation of intracellular sodium ([Na^+^]_i_) was the core driving factor. ^23^Na MRI was used to detect the therapeutic effects of the Na^+^ dependent Na^+^/H^+^ exchanger (NHE1) inhibitor cariporide and the voltage-gated Na^+^ channels (VGSC) inhibitor eslicarbazepine acetate (ESL), neither of which reduced tumor [Na^+^]. This suggests that single-targeting of sodium channels is insufficient to reverse sodium accumulation. The elevation of [Na^+^]_i_ may be co-regulated by multiple sodium transporters, and a combined targeting strategy is required. Targeting the mechanisms underlying the elevation of [Na^+^]_i_ rather than simply inhibiting sodium channels may serve as a novel therapeutic target, while ^23^Na MRI can be used for screening effective drugs (Gast et al., 2023; James et al., 2022; Smith et al., 2025). Additionally, targeting metabolic immune crosstalk, such as reversing lactate-driven PD-1 resistance with LDHA inhibitors, holds promise for overcoming immune evasion (Chen et al., 2022; Li et al., 2023). Furthermore, nanoparticle-delivered agents offer tumor-specific delivery to mitigate toxicity (Swetha & Roy, 2018). Tumor metabolic reprogramming distinguishes the TME from other tissues in the body, like the acid microenvironment caused by Warburg effect, thereby making it possible to delivery drug through tumor metabolic targeting. Based on this, researchers have developed a lactate-responsive drug carrier based on enzyme-assisted Janus mesoporous silica nanoparticles. Lactate oxidase is lactate-responsive, functioning by catalyzing the oxidation of lactate to produce pyruvate and hydrogen peroxide (H_2_O_2_). The production of H_2_O_2_in tumor tissues triggers the self-immolation reaction of arylboronate, leading to the uncapping of mesoporous silica particles, and thus achieving drug release and tumor cell killing (Zhang et al., 2025b). In addition, nanoparticle can achieve combined targeting strategy, which is examplified by the co-delivery of doxorubicin and resveratrol. This has shown significant cytotoxicity on breast cancer cells, which is originally doxorubicin-resistant. Such NP can transport the drug precisely into the targeted nucleus, inducing apoptosis through the down-regulation of B cell lymphoma-2 (Bcl-2) and NF-*κ*B (nuclear factor kappa B) expression, as well as through the inhibition of originally upregulated efflux transporter P-gp (P-glycoprotein) and ATP-binding cassette protein, multidrug resistance protein 1 (MRP-1) and breast cancer resistance protein (BCRP) (Yao et al., 2020; Zhao et al., 2016).

In summary, this review underscores the pivotal role of metabolic reprogramming as a central mechanism underlying drug resistance in cancer. Through alterations in pathways from aerobic glycolysis, lactate metabolism, glutamine addiction, lipid synthesis, mitochondrial adaptation, to ion metabolism, tumor cells not only meet their biosynthetic and aberrant energetic demands but also develop resilience against conventional therapies. The interplay between oncogenic signaling, TME, and other molecules further complicates therapeutic outcomes, often leading to treatment failure and poor prognosis. However, these metabolic vulnerabilities also present promising targets for intervention. Emerging strategies, including inhibitors of key metabolic enzymes, modulators of nutrient transporters, and combination therapies targeting multiple signaling pathways and even immune pathways, hold significant potential to overcome resistance and improve clinical outcomes. Future research should focus on elucidating the context-specific roles of metabolic adaptations across different cancer types and stages, optimizing therapeutic strategies to minimize toxicity. Ultimately, integrating metabolic therapeutics into the framework of precision oncology may pave the way for more durable and effective cancer treatments.

## Additional Information and Declarations

### Data Availability

The following information was supplied regarding data availability: This is a literature review.
